# CD4 T Cells in *Mycobacterium tuberculosis* and *Schistosoma mansoni* Co-infected Individuals Maintain Functional TH1 Responses

**DOI:** 10.3389/fimmu.2020.00127

**Published:** 2020-02-07

**Authors:** Taryn A. McLaughlin, Jeremiah Khayumbi, Joshua Ongalo, Joan Tonui, Angela Campbell, Salim Allana, Samuel Gurrion Ouma, Felix Hayara Odhiambo, Neel R. Gandhi, Cheryl L. Day

**Affiliations:** ^1^Emory Vaccine Center, Emory University, Atlanta, GA, United States; ^2^Center for Global Health Research, Kenya Medical Research Institute, Kisumu, Kenya; ^3^Department of Epidemiology, Rollins School of Public Health, Emory University, Atlanta, GA, United States; ^4^Division of Infectious Diseases, Department of Medicine, Emory University School of Medicine, Atlanta, GA, United States; ^5^Department of Microbiology & Immunology, Emory University School of Medicine, Atlanta, GA, United States

**Keywords:** helminth, *Schistosoma mansoni*, *Mycobacterium tuberculosis*, LTBI, active TB disease, CD4 T cell, TH1, TH2

## Abstract

*Mycobacterium tuberculosis* (Mtb) is a serious public health concern, infecting a quarter of the world and leading to 10 million cases of tuberculosis (TB) disease and 1. 5 million deaths annually. An effective type 1 CD4 T cell (TH1) immune response is necessary to control Mtb infection and defining factors that modulate Mtb-specific TH1 immunity is important to better define immune correlates of protection in Mtb infection. Helminths stimulate type 2 (TH2) immune responses, which antagonize TH1 cells. As such, we sought to evaluate whether co-infection with the parasitic helminth *Schistosoma mansoni* (SM) modifies CD4 T cell lineage profiles in a cohort of HIV-uninfected adults in Kisumu, Kenya. Individuals were categorized into six groups by Mtb and SM infection status: healthy controls (HC), latent Mtb infection (LTBI) and active tuberculosis (TB), with or without concomitant SM infection. We utilized flow cytometry to evaluate the TH1/TH2 functional and phenotypic lineage state of total CD4 T cells, as well as CD4 T cells specific for the Mtb antigens CFP-10 and ESAT-6. Total CD4 T cell lineage profiles were similar between SM^+^ and SM^−^ individuals in all Mtb infection groups. Furthermore, in both LTBI and TB groups, SM infection did not impair Mtb-specific TH1 cytokine production. In fact, SM^+^ LTBI individuals had higher frequencies of IFNγ^+^ Mtb-specific CD4 T cells than SM^−^ LTBI individuals. Mtb-specific CD4 T cells were characterized by expression of both classical TH1 markers, CXCR3 and T-bet, and TH2 markers, CCR4, and GATA3. The expression of these markers was similar between SM^+^ and SM^−^ individuals with LTBI. However, SM^+^ individuals with active TB had significantly higher frequencies of GATA3^+^ CCR4^+^ TH1 cytokine^+^ Mtb-specific CD4 T cells, compared with SM^−^ TB individuals. Together, these data indicate that Mtb-specific TH1 cytokine production capacity is maintained in SM-infected individuals, and that Mtb-specific TH1 cytokine^+^ CD4 T cells can express both TH1 and TH2 markers. In high pathogen burden settings where co-infection is common and reoccurring, plasticity of antigen-specific CD4 T cell responses may be important in preserving Mtb-specific TH1 responses.

## Introduction

Despite advances in care in the past decades, tuberculosis (TB) disease is currently the leading cause of death due a single infectious agent. Nearly 25% of the world's population is infected with the bacteria that causes the disease, *Mycobacterium tuberculosis* (Mtb) (1). Infection with Mtb leads to a spectrum of clinical states ranging from complete clearance, to latent infection (LTBI), to active TB disease (2). The immunological states associated with these differences have not been completely defined, however it is clear that CD4 T cells are necessary to control Mtb infection (3, 4). Furthermore, T cells must be capable of producing type 1 (TH1) cytokines, such as IFNγ and TNFα, which have been shown to be critical in the control of Mtb (5–7).

Co-infections, such as with HIV, and comorbidities, such as diabetes, are known to influence Mtb infection outcomes (1). In addition, infections with numerous helminth species are known to modulate the immune response in a variety of ways. Helminths can directly impair the immune system through the secretion of helminth-derived molecules that act on host immune cells and limit or alter their effector functions (8). Helminths also indirectly impact the immune system by inducing a strongly TH2 polarizing environment that primes immune responses to bystander antigens (9, 10). Both these immune modulation strategies result in systemic immune dysregulation and have long term consequences for immune cell function and disease outcomes. Due to the overlapping geographic distributions of TB burden and helminth infections (11, 12), determining the impact of helminths on Mtb immunity is important in determining correlates of protection against Mtb infection as well as against the development of TB disease. As such, many have investigated this phenomenon and reported differing conclusions. A number of studies in humans have demonstrated that both filarial worms and the soil transmitted helminths *Srongyloides stercoralis* and hookworm can globally dysregulate the immune response to Mtb (13–17). Indeed, all three types of worm have been shown to skew Mtb-specific immune responses by limiting TH1 cytokine production and increasing TH2 cytokine production in response to Mtb antigens in individuals with LTBI (18–21); moreover, treatment of helminth infections in people with LTBI has been shown to result in increased the frequencies of Mtb-specific IFNγ^+^ CD4 T cells (22). Others, however, have shown no demonstrable effect on either immunity to Mtb or disease outcomes during co-infection with helminths, including filarial worms and hookworm (23, 24). One recent study even reported an increased ability to control Mtb growth in individuals infected with hookworm (25). This variation is particularly evident in a recent meta-analysis of epidemiological studies of individuals co-infected with Mtb and helminths. The report indicates that both the prevalence rate of co-infection as well as the measured associations between infections varies greatly between studies as well as between helminth species (26). The outcome of helminth co-infection on the immune response to Mtb is likely specific to both the helminth species, as well as the Mtb infection status of the individuals being studied.

Schistosomiasis, the disease caused by schistosome worms such as *Schistosoma mansoni* (SM), is estimated to affect 240 million people globally and is the second most common form of helminthiasis (27, 28). Approximately 90% of affected individuals live in Sub-Saharan Africa and while not normally not a fatal infection, the mortality rates for schistosome infections in Sub-Saharan Africa are estimated to be 280,000 per year (29). Similar to both filarial worms and soil-transmitted helminths, SM has a complex life cycle. It enters a human host as a cercariae and then matures as it migrates throughout the body. Eventually adult worms take up residence in the portal vein and release eggs into the circulation, which are able to pass into the lumen of the gastrointestinal tract and are subsequently released in stool (30). Unlike other helminth species, however, the immune response to SM is stage-dependent. Early stages of the worm life cycle stimulate a mixed TH1/TH17 response, which only gives rise to a TH2 response upon egg secretion (31). As such, the impact of SM on Mtb-specific immune responses may be quite different from what has previously been reported in individuals co-infected with Mtb and either filarial or soil transmitted helminths. Despite this, the impact of co-infection with SM on Mtb-specific TH1 responses has not been thoroughly investigated in humans. One study in mice reported that SM infection impairs Mtb-specific TH1 CD4 T cell responses and increases arginase-1 expressing macrophages in type 2 granulomas (32). Furthermore, data regarding the impact of SM on Mtb-specific immune responses in general is conflicting. In mice, SM has no impact on Mtb-specific cytokine production or antibody responses generated by DNA vaccination (33, 34); however, it impairs Mtb-specific cytokine production following BCG vaccination and results in higher CFU of both BCG and Mtb (35, 36). In humans, SM infection does not impair the generation of Mtb-specific T and B cells by the TB candidate vaccine MVA85A (37). *In vitro* studies of human PBMCs exposed to SM antigens have demonstrated skewing of Mtb-specific CD4 T cells from a TH1 to a TH2 response; however, human monocyte-derived macrophages exposed to the same antigens have produced contradictory results with one study showing enhanced control and another showing impaired control of Mtb replication *in vitro* (38, 39). It therefore remains unclear what impact, if any, co-infection with SM has on Mtb-specific immune responses, particularly in humans.

We sought to test the hypothesis that SM infection modifies the lineage profile of Mtb-specific CD4 T cells away from a dominant TH1 toward a TH2 phenotype. TH1 cells are characterized by their production of key TH1 cytokines such as IFNγ and their expression of specific lineage markers, namely the transcription factor T-bet and/or the chemokine receptor CXCR3 (40–42). TH2 cells can be similarly characterized by their production of TH2 cytokines such as IL-4 and their expression of the transcription factor GATA3 and/or the chemokine receptor CCR4 (40–42). While CD4 T cell subsets have been defined by canonical transcription factors and chemokine receptors, there is growing appreciation for the variability and plasticity of CD4 T cells (43–46). As such, we performed a comprehensive analysis of the lineage state of CD4 T cells in a well-characterized cohort of Kenyan adults representing a spectrum of Mtb infection and disease. We enrolled individuals in groups defined by Mtb and SM infection status: healthy controls (HC), latent TB infection (LTBI), and active TB (TB), both with and without SM infection. In each group we examined CD4 T cells for the expression of cytokines, transcription factors, and chemokine receptors associated with TH1 and TH2 lineage commitment simultaneously. This allowed us to evaluate CD4 T cell lineage using both phenotypic and functional readouts; moreover, we measured co-expression of these markers to determine the variability and plasticity of the Mtb-specific CD4 T cell repertoire in the setting of human Mtb and SM co-infection.

## Materials and Methods

### Study Population and Sample Collection

Participants 18–81 years old were recruited in Kisumu, Kenya as described previously (47). Healthy asymptomatic individuals with no previous history of TB disease or treatment were evaluated by QuantiFERON®-TB Gold In-Tube (QFT) assay: those with a negative QFT result (QFT^−^; TB Ag-Nil < 0.35 IU IFNγ/mL) were defined as healthy controls (HC); those with a positive QFT result (TB Ag-Nil > 0.35 IU IFNγ/mL) were defined as having latent Mtb infection (LTBI). All HC and LTBI participants had normal chest x-rays. Patients with drug-sensitive active pulmonary tuberculosis disease (TB) were symptomatic individuals with a positive GeneXpert MTB/RIF result and a positive culture for Mtb growth. Blood was collected from individuals with active TB within the first 7 days of initiating the standard 6 months course of TB treatment, which was provided according to Kenyan national health guidelines. Chest x-rays were not performed on individuals with active TB. All participants are presumed to be BCG vaccinated due to the Kenyan policy of BCG vaccination at birth and high BCG coverage rates throughout Kenya (48, 49). *Schistosoma mansoni* (SM) infection was determined using standard Kato Katz microscopy. Briefly, two thick Kato Katz smears were prepared from stool samples collected on two separate days. Slides were analyzed by experienced lab technicians who recorded the presence of SM eggs as well as the number of eggs counted. Participants were excluded if eggs belonging to other helminth species including *Ascaris lumbricoides, Trichuris trichuria* and hookworm were identified. Participants were not tested for lymphatic filariasis since it is not endemic in western Kenya (11). Other exclusion criteria included: pregnancy, hemoglobin value of <7.0 g/dl, HIV infection, and positive rapid malaria test. Blood was collected from patients in sodium heparin Vacutainer® CPT™ Mononuclear Cell Preparation Tubes (BD Biosciences). PBMC were isolated by density centrifugation, cryopreserved in freezing medium (50% RPMI 1640 + 40% heat-inactivated fetal calf serum [FCS] + 10% DMSO), and stored in LN_2_ until use.

### Ethics Statement

This study was conducted in accordance with the principles expressed in the Declaration of Helsinki. All participants gave written informed consent for the study, which was approved by the KEMRI/CDC Scientific and Ethics Review Unit and the Emory University Institutional Review Board.

### Antigens

This study utilized peptide pools of the immunodominant Mtb antigens CFP-10 and ESAT-6 (1 μg/ml of each peptide). Pools of 15-mer overlapping peptides spanning the full-length sequences of CFP-10 and ESAT-6 were obtained through BEI Resources, NIAID, NIH (catalog numbers NR-50712 and NR-50711, respectively). For the overnight intracellular cytokine staining assay, phorbol 12-myristate 13-acetate, (PMA, 50 ng/mL, Adipogen) and ionomycin (1 μg/mL, Cayman Chemical) were used as a positive control. In the 5 days proliferation assay, Staphylococcal enterotoxin B (SEB; 1 μg/mL, Toxin Technology, Inc.) was used as a positive control for proliferation while PMA and ionomycin were used to induce cytokine production for the final 5 h of the proliferation assay.

### Antibodies

The following human monoclonal fluorescently-conjugated antibodies were used in this study: anti-CD3 BV605 (clone OKT-3), anti-CD4 BV570 (clone RPA-T4), anti-CCR4 BV421 (clone L291H4), anti-T-bet PE-Cy7 (clone 4B10), anti-TNFα Alexa Flour 647 (clone Mab11), and anti-IL-4 PE-Dazzle594 (clone MP4-25D2), all from BioLegend; anti-CD4 BV786 (clone SK3), anti-CD8 PerCP-Cy5.5 (clone SK-1), anti-CXCR3 BV711 (clone 1C6), anti-GATA3 PE (clone L50-823), and anti-IFN-γ Alexa Fluor 700 (clone B27), all from BD Biosciences; and anti-IL-13 FITC (clone 85BRD) from eBiosciences.

### PBMC Overnight Intracellular Cytokine Staining (ICS) Assay

Cryopreserved PBMCs were thawed in a 37°C water bath and immediately added to RPMI 1640 (Cellgro) containing deoxyribonuclease I (DNase, 10 μg/ml, Sigma-Aldrich). Cells were washed twice in RPMI and then suspended in R10 media (RPMI 1640 supplemented with 10% heat-inactivated fetal calf serum [FCS], 100 U/ml penicillin, 100 μg/ml streptomycin, and 2 mM L-glutamine) and rested for a minimum of 3 h at 37°C and 5% CO_2_ before the addition of antigens (described above). Cells incubated in R10 media alone served as a negative control. After 3 hrs, brefeldin A (10 μg/ml; Sigma-Aldrich) and monensin (1x, BioLegend) were added and the incubation continued for an additional 15 hrs.

### PBMC Proliferation Assay

Cryopreserved PBMCs were thawed, washed in PBS containing deoxyribonuclease I (DNase, 10 μg/ml, Sigma-Aldrich). Cells were washed twice in PBS and then labeled with 0.5 μg/ml CellTrace™ Oregon Green® 488 carboxylic acid diacetate, succinimidyl ester (OG; Life Technologies). Cells were washed once more with PBS and resuspended in R10 media (RPMI 1640 supplemented with 10% heat-inactivated human serum, 100 U/ml penicillin, 100 μg/ml streptomycin, and 2 mM L-glutamine) containing recombinant human IL-2 (10 Units/mL, obtained through the NIH AIDS Reagent Program, Division of AIDS, NIAID, NIH) (50). Cells were plated in 96-well plates and incubated for 5 days in a 37°C incubator with 5% CO_2_. On day 5, 75 μl of cell culture supernatant per well were removed and stored for Luminex analysis (see below). Cells were then resuspended in 250 μL R10 media. With the exception of the negative control (wells containing cells in media alone) cells were re-stimulated with PMA and ionomycin (described above) and treated with brefeldin A (10 μg/ml; Sigma-Aldrich) and monensin (1x, Biolegend) for 5 hrs at 37°C to determine the cytokine capacity of proliferating CD4 T cells.

### Antibody Staining for Flow Cytometry

Following stimulation, cells were washed with PBS and stained with the Fixable Viability Dye Zombie Near-IR (BioLegend) for 15 min at room temperature. Samples were then surface stained for 30 min at room temperature. For the ICS assay this included: anti-CD3 BV605, anti-CD4 BV570, anti-CD8 PerCP-Cy5.5, anti-CCR4 BV421, and anti-CXCR3 BV711. For the proliferation assay: anti-CD3 BV605, CD4 BV786, anti-CD8 PerCP-Cy5.5, anti-CCR4 BV421, and anti-CXCR3 BV711. Following the surface stain, cells were fixed and permeabilized on ice for 1 h using the FoxP3 Transcription Staining Buffer Set (eBioscience). Cells were then stained for intracellular markers on ice for 40 min. For the ICS assay this included: anti-T-bet PE-Cy7, anti-GATA3 PE, anti-IFN-γ Alexa Fluor 700, anti-TNFα Alexa Flour 647, anti-IL-4 PE-Dazzle594, and anti-IL-13 FITC. For the proliferation assay this included: anti-T-bet PE-Cy7, anti-GATA3 PE, anti-IFN-γ Alexa Fluor 700, anti-TNFα Alexa Flour 647, and anti-IL-4 PE-Dazzle594. Finally, cells were washed in permeabilization buffer and resuspended in PBS. Samples were acquired using a BD LSR II flow cytometer.

### Luminex

Cell culture supernatants from each well were harvested on day 5 of the proliferation assay described above. Supernatants were frozen and stored at −80°C until use. The following cytokines were measured using a customized *R*&*D* Human Magnetic Luminex Assay kit (Biotechne) in batched analyses, following the manufacturer's instructions: IFNγ, TNFα, IL-21, IL-22, IL-17, IL-4, IL-5, IL-10, IL-13. The mean fluorescence intensity was read for each cytokine using a Luminex MAGPIX® system with xPONENT® software (Version 4.2) and analyzed using MILLIPLEX® Analyst 5.1 Software. Cytokine data from antigen-stimulated wells are reported after subtraction of corresponding cytokine levels in the negative control wells.

### Data Analysis

Flow cytometry data were analyzed using FlowJo version 9.6.4 (BD). Compensation was calculated using single-stained anti-mouse Ig,κ CompBeads (BD Biosciences). Single cells were gated by plotting forward scatter-area vs. forward scatter-height; lymphocytes were gated based on morphological characteristics. Viable cells were defined as Zombie Near-IR^lo^ cells. CD4 T cells were defined as CD3^+^CD4^+^CD8^−^ lymphocytes. Antigen-specific CD4 T cell populations were defined as cells producing cytokines (IFN-γ, TNF-α, IL-4, and/or IL-13) after stimulation with antigen. Proliferating cells were defined as those with low expression of the cytosolic dye Oregon Green (OG^lo^). The flow cytometry gating strategy is indicated in Figure S1. Responses were evaluated using the mixture models for single-cell assays (MIMOSA) method to determine positivity using a Markov Chain Monte Carlo algorithm with a prior of 0.01% in the ICS assay and a prior of 1% in the proliferation assay (51). Samples with a probability of response >70% and a false discovery rate (fdr/q-value) <3% were considered positive. Phenotypic analysis of lineage marker expression on antigen-specific CD4 T cells was restricted to individuals who met the above criteria for a positive response.

### Statistical Analysis

R programming software was used to perform all statistical analyses. Differences between SM^+^ and SM^−^ individuals within each stratum of Mtb infection were evaluated using a non-parametric Mann-Whitney test. Differences between three or more groups were evaluated using a non-parametric Kruskal-Wallis test and corrected for multiple comparisons using the Bonferroni method. Correlations were evaluated using a non-parametric Spearman rank correlation. *P*-values < 0.05 were considered significant. Graphs were created using the R package ggplot2 and statistics were performed using the stats package.

## Results

### Study Participants

Participants were recruited and enrolled in Kisumu, Kenya and categorized into six groups based on their Mtb and SM infection status: HC, LTBI, and TB, with or without concomitant SM infection (Table 1). Within each Mtb infection group, SM^+^ and SM^−^ individuals had similar demographic profiles with two exceptions. First, there were more females in the LTBI SM^−^ group than the LTBI SM^+^ group. In addition, amongst active TB individuals, SM^+^ individuals were older than those that were SM^−^.

**Table 1:** Characteristics of study participants.

	**Healthy controls (HC)**		**Latent Mtb infection (LTBI)**		**Active TB disease (TB)**	
	**SM^−^** ***N = 24***	**SM^**+**^** ***N = 13***	**SM^−^** ***N = 26***	**SM^**+**^** ***N = 24***	**SM^−^** ***N = 26***	**SM^**+**^** ***N = 16***
Age (years)^a^	24	25	36	34	28	40^b^
(IQR)	(21–28)	(21–32)	(23–55)	(25–38)	(21–35)	(26–45)
sex: (%M)	37.5	23.1	23.1	58.3^c^	69.2	75.0
(%F)	62.5	76.9	76.9	41.7	30.8	25.0
Hemoglobin g/dl^a^	13.5	13.6	12.9	14.0	12.1	11.7
(IQR)	(12.6–14.7)	(12.6–14.4)	(11.3–14.1)	(12.4–15.0)	(10.7–14.1)	(10.8–12.2)
SM eggs/gram^a^	0	36	0	150	0	48
(IQR)		(12–120)		(36–333)		(21–87)
QFT IU/mL^a^	0.00	0.00	7.94	9.11	ND^d^	ND^d^
(IQR)	(0.00–0.05)	(0.00–0.10)	(2.91–9.25)	(5.36–9.54)		

a *Value denotes median*.

b *p < 0.05, compared with TB/SM^−^*.

c *p < 0.05, compared with LTBI/SM^−^*.

d *Not done*.

*IQR, interquartile range*.

### Similar Frequencies of CD4 T Cells Express TH1 and TH2 Lineage Markers in SM^+^ and SM^−^ Individuals Independent of Mtb Infection Status

**Figure 1:**
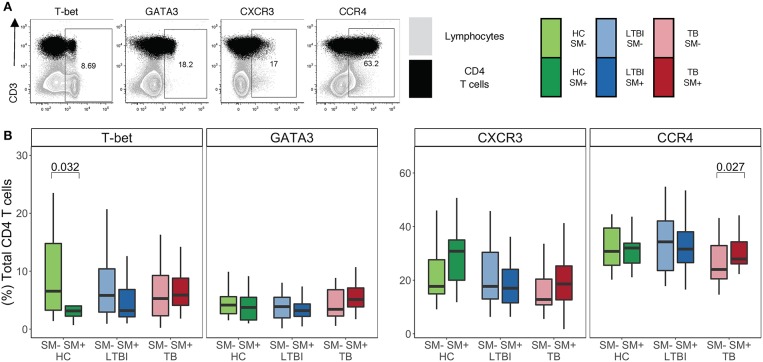
Similar frequencies of CD4 T cells express TH1 and TH2 lineage markers in SM^+^ and SM^−^ individuals, independent of Mtb infection status. Expression of transcription factors and chemokine receptors was measured by flow cytometry using PBMC samples obtained from individuals in each of six groups defined by Mtb and SM infection status: HC, LTBI, and TB, with or without concomitant SM infection (HC SM^−^, *n* = 24; HC SM^+^, *n* = 13; LTBI SM^−^, *n* = 25; LTBI SM^+^, *n* = 25; TB SM^−^, *n* = 25; TB SM^+^, *n* = 15). **(A)** Representative flow cytometry data from an SM^+^ individual with active TB. Plots show cells gated on live CD3^+^CD4^+^CD8^−^ lymphocytes in black dots overlaying live lymphocytes in gray. **(B)** Frequency of indicated transcription factor^+^ and chemokine receptor^+^ amongst total CD4 T cells. Boxes represent the median and interquartile ranges; whiskers represent the 1.5^*^IQR. Differences in the frequency of each lineage marker^+^ CD4 T cell population between SM^+^ and SM^−^ individuals were assessed using a Mann Whitney U test.

Infection with helminths is associated with skewing of the immune system to a TH2 CD4 T cell response (18–21). To investigate the impact of SM infection on the CD4 T cell repertoire, we utilized flow cytometry to measure the expression of TH1 and TH2 lineage markers in total CD4 T cells (Figure 1A). This analysis was done within each stratum of Mtb infection to account for a possible differential impact of SM within diverse infection states. We first measured the frequency of CD4 T cells expressing the canonical TH1 and TH2 transcription factors, T-bet and GATA3, respectively (Figure 1B). Only HC exhibited significant differences in transcription factor expression, with SM^+^ individuals having a lower frequency of T-bet^+^ CD4 T cells than SM^−^ counterparts. This difference is not observed in LTBI or TB groups. Importantly, the frequency of transcription factor^+^ CD4 T cells was not dominated by either transcription factor, demonstrating a balance of these responses in the total CD4 T cell repertoire. We then measured the expression of the chemokine receptors CXCR3 and CCR4, which are associated with TH1 and TH2 lineage commitment, respectively (Figure 1B). CXCR3 expression was similar across groups whereas CCR4 expression was higher in SM^+^ TB individuals compared to SM^−^ TB individuals. These data indicate that across Mtb infection states, SM does not substantially alter the baseline lineage phenotype of total CD4 T cells of individuals.

### SM Infection Does Not Bias the Capacity of CD4 T Cells to Produce TH1 and TH2 Cytokines

**Figure 2:**
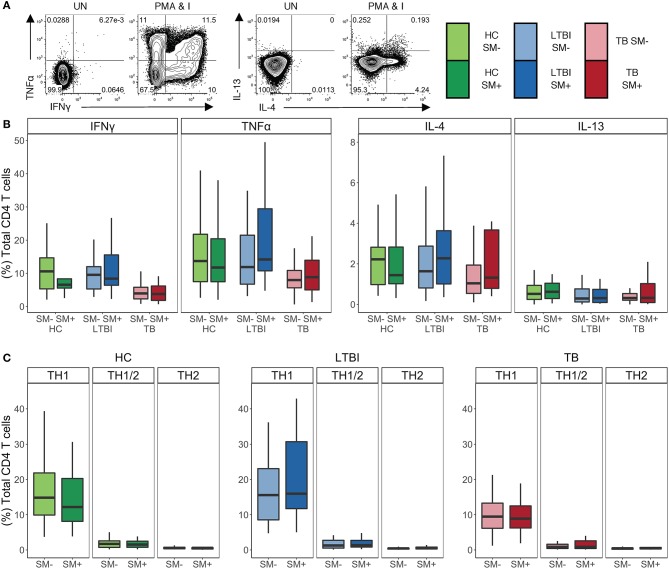
SM^+^ and SM^−^ individuals have similar frequencies of TH1 and TH2 cytokine^+^ CD4 T cells, with TH1 cytokines being the dominant response independent of SM and Mtb infection status. PBMC samples obtained from individuals in each of six groups defined by TB and SM infection status were incubated for 18 h in media alone (negative control) or stimulated with PMA and ionomycin. Intracellular expression of IFNγ, TNFα, IL-4, and IL-13 was measured by flow cytometry (HC SM^−^, *n* = 24; HC SM^+^, *n* = 13; LTBI SM^−^, *n* = 25; LTBI SM^+^, *n* = 25; TB SM^−^, *n* = 25; TB SM^+^, *n* = 15). **(A)** Representative flow cytometry data from an SM^+^ HC. Plots are shown gated on live CD3^+^CD4^+^CD8^−^ lymphocytes from the unstimulated (UN) and stimulated condition. **(B)** Frequencies of total CD4 T cells expressing each indicated cytokine. **(C)** Cytokine^+^ cells were aggregated by T cell lineage (Figure S2) and the frequency of each group was reported. Data are shown after subtraction of background cytokine production in the unstimulated negative control condition. Boxes represent the median and interquartile ranges; whiskers represent the 1.5*IQR. Differences in the frequency of each cytokine^+^ CD4 T cell population between SM^+^ and SM^−^ individuals were assessed using a Mann Whitney U test. Differences in the frequencies of TH1, TH1/2, and TH2 CD4 T cells within each group were evaluated using a Kruskal Wallis test. TH1 cytokine frequencies were statistically higher than the both TH1/2 and TH2 frequencies after applying the Bonferroni correction for multiple comparisons.

In addition to phenotypic markers, CD4 T cell polarization can be evaluated by cytokine production. As such, we sought to investigate whether there were differences in the cytokine profiles of CD4 T cells in individuals infected with SM. To this end, we stimulated PBMC with PMA and ionomycin and then performed intracellular cytokine staining (ICS) for TH1 cytokines IFNγ and TNFα, as well as TH2 cytokines IL-4 and IL-13 (Figure 2A). We used flow cytometry to quantify the production of these cytokines individually as well as in combination. There were no differences in the total frequency of CD4 T cells producing any of the four cytokines between SM^+^ and SM^−^ individuals across Mtb infection groups (Figure 2B). Furthermore, the median fluorescence intensity (MFI) of each cytokine measured did not differ between SM^+^ and SM^−^ individuals (data not shown). Further analysis was done to evaluate combinations of cytokines being produced using a Boolean gating strategy. In all Mtb infection groups, the frequency of each combination of cytokines was similar between SM^+^ and SM^−^ individuals (Figure S2).

To evaluate the functional lineage state of CD4 T cells, we collapsed these Boolean gates into three categories of cells: TH1, TH2, and TH1/2 (Figure S2). Briefly, TH1 cells were defined as cells producing IFNγ and/or TNFα but not IL-4 or IL-13, TH2 cells were defined as cells producing IL-4 and/or IL-13 but not IFNγ or TNFα, and TH1/2 cells were defined as cells producing a combination of IFNγ or TNFα and IL-4 or IL-13. No significant differences in the frequencies of these cell subsets were observed between SM^+^ and SM^−^ individuals in all three Mtb infection groups (Figure 2C). Furthermore, in all of the participant groups, the frequency of TH1 cytokine^+^ CD4 T cells was significantly higher than that of either TH2 or TH1/2 cytokine^+^ CD4 T cells. Together, these data provide further evidence that SM does not modify the TH1 and TH2 cytokine production capacity of CD4 T cells across Mtb infection groups.

### TH1 Cytokine^+^ CD4 T Cells Express Low to Moderate Levels of TH1 Lineage Markers While TH2 cytokine^+^ CD4 T Cells Express High Levels of TH2 Lineage Markers Independent of SM and Mtb Infection Status

**Figure 3:**
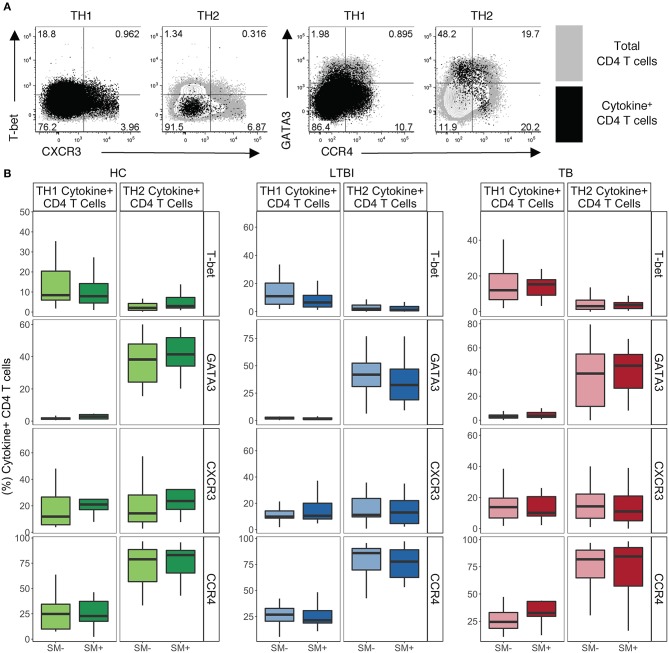
TH1 cytokine^+^ cells express low levels of TH1 lineage markers while TH2 cytokine^+^ CD4 T cells express high levels of TH2 lineage markers independent of SM and TB infection status. PBMC samples obtained from individuals in each of six groups defined by Mtb and SM infection were incubated for 18 h in media alone (negative control) or stimulated with PMA and ionomycin. Intracellular expression of IFN-γ, TNF-α, IL-4, and IL-13 was evaluated by flow cytometry and cytokine^+^ cells were aggregated by functional T cell lineage (Figure S2). Samples meeting the criteria for a positive response (see Materials and Methods) were evaluated for expression of lineage specific transcription factors and chemokine receptors by flow cytometry. **(A)** Representative flow cytometry data from an SM^−^ HC. Plots show either TH1 cytokine^+^ or TH2 cytokine^+^ live CD3^+^CD4^+^CD8^−^ lymphocytes in black dots overlaying total live CD3^+^CD4^+^ lymphocytes in gray. **(B)** Frequency of indicated transcription factor^+^ and chemokine receptor^+^ amongst TH1 cytokine^+^ or TH2 cytokine^+^ CD4 T cells in HC (SM^−^, *n* = 24; SM^+^, *n* = 13), LTBI (SM^−^, *n* = 25; SM^+^, *n* = 25), and TB (SM^−^, *n* = 25; SM^+^, *n* = 14). Boxes represent the median and interquartile ranges; whiskers represent the 1.5*IQR. Differences in the frequency of each lineage marker^+^ CD4 T cell population between SM^+^ and SM^−^ individuals were assessed using a Mann Whitney *U*-test.

We next analyzed expression of lineage specific markers on cytokine^+^ CD4 T cells following stimulation with PMA and ionomycin. We utilized our functional lineage gating definitions (Figure S2) to define TH1 and TH2 cells and then used MIMOSA (see materials and methods) to evaluate samples with a positive cytokine^+^ response for expression of T-bet, GATA3, CXCR3, and CCR4 on each cytokine subset of CD4 T cells (Figure 3A).

In HC, LTBI, and TB groups, TH1 cytokine^+^ CD4 T cells had intermediate expression of TH1 lineage markers (Figure 3B). As expected, TH1 cytokine^+^ cells were almost exclusively GATA3^−^, however only low to moderate frequencies were T-bet^+^. In addition, TH1 cytokine^+^ cells had low to moderate frequencies of CXCR3^+^ and CCR4^+^ cells. These expression profiles of TH1 cytokine^+^ CD4 T cells did not differ by SM or Mtb infection status.

By contrast, TH2 cytokine^+^ CD4 T cells have more distinct TH2-like lineage marker expression in HC, LTBI and TB groups (Figure 3B). Approximately half the TH2 cytokine^+^ cells in each group were GATA3^+^, with only a minority of cells expressing T-bet. TH2 cytokine^+^ cells were also predominantly CCR4^+^ with low frequencies of CXCR3^+^ cells. These expression profiles of TH2 cytokine^+^ CD4 T cells did not differ by SM or Mtb infection status. These data demonstrate that CD4 T cells, as defined by TH1 and TH2 cytokine production, do not strictly adhere to canonical expression patterns of TH1 and TH2 phenotypic markers.

### LTBI Individuals Co-infected With SM Have Higher Frequencies of TH1 Cytokine^+^ Mtb-Specific CD4 T Cells, Compared With SM^−^ Individuals With LTBI

**Figure 4:**
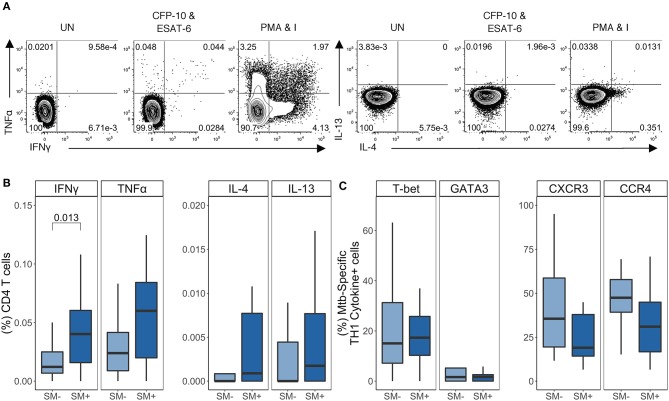
SM^+^ LTBI individuals have higher frequencies of TH1 cytokine^+^ Mtb-specific CD4 T cells, which express both TH1 and TH2 lineage markers. PBMC samples obtained from SM^+^ and SM^−^ LTBI individuals were incubated for 18 h in media alone (negative control), PMA and ionomycin (positive control), or CFP-10 and ESAT-6 peptide pools. Intracellular expression of IFN-γ, TNF-α, IL-4, and IL-13 was measured by flow cytometry. **(A)** Representative flow cytometry data from an SM^+^ individual. Plots are shown gated on live CD3^+^CD4^+^CD8^−^ lymphocytes from the unstimulated and stimulated conditions. **(B)** Frequency of each subset of TH1 cytokine^+^ and TH2 cytokine^+^ CD4 T cells (SM^−^, *n* = 24; SM^+^, *n* = 22). Data are shown after subtraction of background cytokine production in the unstimulated negative control condition. **(C)** Samples meeting the criteria for a positive TH1 cytokine response (see Materials and Methods) were evaluated for expression of lineage specific transcription factors and chemokine receptors by flow cytometry. The frequency of indicated transcription factor^+^ and chemokine receptor^+^ TH1 cytokine^+^ CD4 T cells are reported (SM^−^, *n* = 16; SM^+^, *n* = 18). Boxes represent the median and interquartile ranges; whiskers represent the 1.5*IQR. Differences in the frequency of each CD4 T cell population between SM^+^ and SM^−^ individuals were assessed using a Mann Whitney *U*-test.

Having established that SM infection does not impair the capacity of CD4 T cells to produce TH1 cytokines, nor does it skew CD4 T cells in general toward a TH2 phenotype, we next evaluated the impact of SM on Mtb-specific CD4 T cell responses in individuals with LTBI. We utilized our ICS assay to measure the frequency of cytokine^+^ CD4 T cells after stimulation of PBMC with CFP-10 and ESAT-6 peptide pools (Figure 4A). The frequency of IFNγ^+^ CD4 T cells was significantly higher in SM^+^ than SM^−^ individuals (Figure 4B). When cytokine production was analyzed across combinations of cytokines, this difference was found to be due to a population of cells co-producing IFNγ and TNFα (Figure S3A). Importantly, there was no difference in Mtb-specific TH2 cytokine production between SM^+^ and SM^−^ individuals (Figure 4B) nor in the MFI of TH2 cytokines (data not shown). Furthermore, TH1 cytokines were the dominant response to CFP-10 and ESAT-6 peptide pools in both groups (Figure S3B). These data indicate that unlike other helminth co-infections, SM infection is associated with increased frequencies of Mtb-specific TH1 cytokine^+^ CD4 T cells amongst individuals with LTBI.

We next evaluated T-bet, GATA3, CXCR3, and CCR4 expression by TH1 cytokine^+^ Mtb-specific CD4 T cells (Figure 4C). Similar to PMA/ionomycin-induced TH1 cytokine^+^ CD4 T cells, Mtb-specific TH1 cytokine^+^ CD4 T cells expressed moderate levels of T-bet. Interestingly, there were also GATA3^+^ TH1 cytokine^+^ cells, though these frequencies were low (<10% of Mtb-specific TH1 cytokine^+^ CD4 T cells) and did not differ by SM infection status (Figure 4C). Further analysis of these Mtb-specific TH1 cytokine^+^ CD4 T cells indicated that they did not co-express GATA3 and T-bet (Figure S3C). Moderate frequencies of Mtb-specific TH1 cytokine^+^ cells expressed CXCR3^+^ and/or CCR4^+^, although this did not differ by SM infection status (Figure 4C and Figure S3C). These data indicate that Mtb-specific CD4 T cells in individuals with LTBI express predominately TH1 cytokines and are not skewed toward a TH2 phenotype in the presence of SM co-infection.

### Individuals With Active TB Disease and SM Co-infection Have Higher Frequ encies of GATA3^+^CCR4^+^ TH1 Cytokine^+^ Mtb-Specific CD4 T Cells

**Figure 5:**
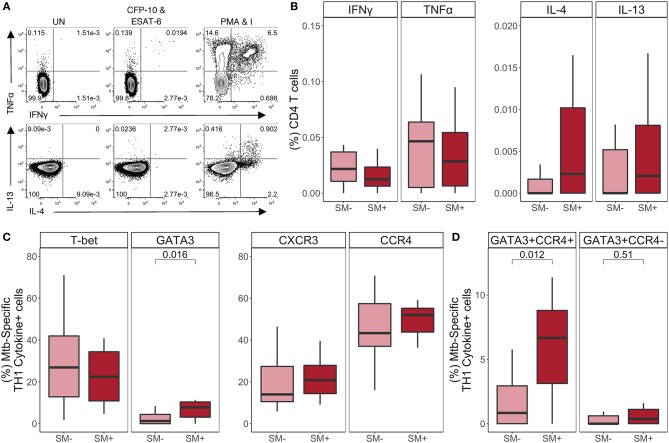
SM^+^ individuals with active TB disease have similar frequencies of TH1 cytokine^+^ Mtb-specific CD4 T cells, but higher expression of GATA3 and CCR4, compared with SM^−^ TB patients. PBMC samples obtained from SM^+^ and SM^−^ TB individuals were incubated for 18 h in media alone (negative control), PMA and ionomycin (positive control), or CFP-10 and ESAT-6 peptide pools. Intracellular expression of IFN-γ, TNF-α, IL-4, and IL-13 was measured by flow cytometry. **(A)** Representative flow cytometry data from an SM^+^ patient with active TB disease. Plots are shown gated on live CD3^+^CD4^+^CD8^−^ lymphocytes from the unstimulated and stimulated conditions. **(B)** Frequency of Mtb-specific CD4 T cells producing each indicated cytokine (SM^−^, *n* = 25; SM^+^, *n* = 15). Data are shown after subtraction of background cytokine production in the unstimulated negative control condition. **(C,D)** Samples meeting the criteria for a positive Mtb-specific TH1 cytokine response (see Materials and Methods) were evaluated for expression of lineage specific transcription factors and chemokine receptors by flow cytometry. The frequency of Mtb-specific TH1 cytokine^+^ CD4 T cells expressing the indicated transcription factor and chemokine receptor **(C)** as well as co-expressing cells **(D)** are reported (SM^−^, *n* = 15; SM^+^, *n* = 9). Boxes represent the median and interquartile ranges; whiskers represent the 1.5*IQR. Differences in the frequency of each CD4 T cell population between SM^+^ and SM^−^ individuals were assessed using a Mann Whitney *U*-test.

We next evaluated the impact of SM on Mtb-specific CD4 T cell responses in individuals with active TB disease (Figure 5A). The total frequency of each cytokine was similar between SM^+^ and SM^−^ individuals (Figure 5B). We confirmed these results by comparing the MFI of each cytokine, which did not differ between SM^+^ and SM^−^ groups (data not shown). Indeed, TH1 cytokines remained the dominant CD4 T cell response to Mtb in both SM^+^ and SM^−^ individuals with active TB disease, and the frequency of these cells did not differ between groups (Figures S4A,B).

We next evaluated T-bet, GATA3, CXCR3, and CCR4 expression by TH1 cytokine^+^ Mtb-specific CD4 T cells (Figure 5C). Consistent with the LTBI group, there were more T-bet^+^ than GATA3^+^ Mtb-specific TH1 cytokine^+^ CD4 T cells; however, while T-bet^+^ frequencies were similar between SM^+^ and SM^−^ individuals, GATA3^+^ frequencies were significantly higher in SM^+^ individuals compared to SM^−^ individuals. The total frequencies of CCR4^+^ and CXCR3^+^ TH1 cytokine^+^ cells did not differ by SM infection (Figure 5C). However, there were significantly higher frequencies of Mtb-specific CD4 T cells co-expressing GATA3 and CCR4 in the SM^+^ TB group, compared with SM^−^ TB group (Figure 5D). When co-expression of these markers was analyzed further, this population was strictly limited to T-bet^−^ cells (Figure S4C). Together these data indicate while Mtb-specific TH1 cytokine production is preserved in SM^+^ individuals with active TB, these TH1 cytokine^+^ cells express higher levels of the canonical TH2 markers GATA-3 and CCR4, compared with Mtb-specific CD4 T cells from SM^−^ TB individuals.

### SM Infection Does Not Significantly Impair CD4 T Cell Proliferative Capacity

**Figure 6:**
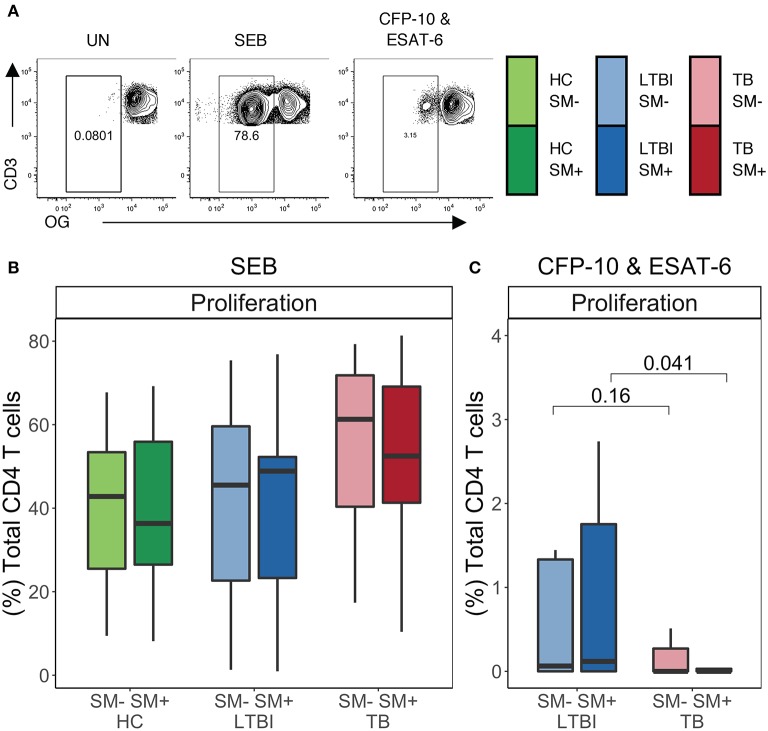
Similar frequencies of CD4 T cells proliferate in SM^+^ and SM^−^ individuals independent of Mtb infection status. Proliferation assays were performed using PBMC samples obtained from individuals in each of six groups defined by Mtb and SM infection status (HC SM^−^, *n* = 24; HC SM^+^, *n* = 13; LTBI SM^−^, *n* = 25; LTBI SM^+^, *n* = 25; TB SM^−^, *n* = 25; TB SM^+^, *n* = 15). Cells were labeled with Oregon Green (OG) and incubated for 5 days under the following conditions: media alone (negative control), SEB (positive control) or CFP-10 and ESAT-6 peptide pools. **(A)** Representative flow cytometry data from an SM^+^ individual with LTBI. Plots show cells gated on live CD3^+^CD4^+^CD8^−^ lymphocytes. **(B,C)** Frequency of OG^lo^ (proliferating) CD4 T cells to SEB and CFP-10 and ESAT-6 peptide pools. Data are shown after subtraction of background proliferation in the unstimulated negative control condition. Boxes represent the median and interquartile ranges; whiskers represent the 1.5*IQR. Differences in the frequency of each CD4 T cell population between SM^+^ and SM^−^ individuals were assessed using a Mann Whitney *U*-test.

To further evaluate the functional capacity of CD4 T cells in the setting of SM infection, we next performed a proliferation assay. PBMC from each group were labeled with the cytosolic dye Oregon Green (OG) and incubated for 5 days either with media alone (negative control), SEB (positive control), or Mtb CFP-10 and ESAT-6 peptide pools. We then measured proliferation via flow cytometry (Figure 6A). No significant differences in CD4 T cell proliferative capacity were observed following stimulation with SEB between SM^+^ and SM^−^ individuals in any of the Mtb infection groups (Figure 6B). We next measured the proliferative capacity of Mtb-specific CD4 T cells in individuals with either LTBI or active TB. There were no statistically significant differences in CD4 T cell proliferation following stimulation with Mtb peptides between SM^+^ and SM^−^ individuals in either group; however, within the SM^+^ groups, TB individuals had markedly lower proliferation in response to Mtb peptides than LTBI individuals (Figure 6C), consistent with previous reports of impaired Mtb-specific CD4 T cell proliferative capacity in individuals with active TB disease (52). These data indicate that TB disease, but not SM infection status, impacts the ability of CD4 T cells to proliferate in response to Mtb peptides.

### Proliferating CD4 T Cells Express TH1 and TH2 Lineage Markers in SM^+^ and SM^−^ LTBI Individuals

**Figure 7:**
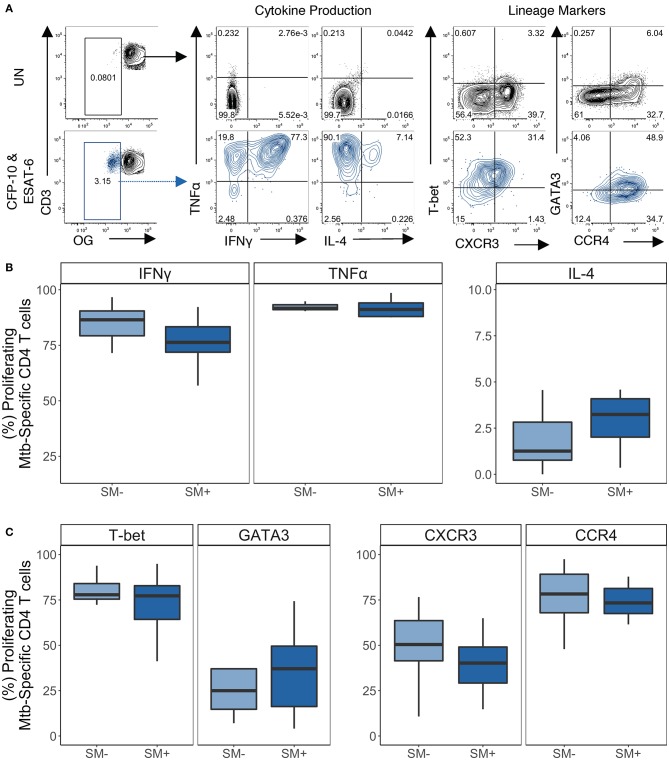
Proliferating Mtb-specific CD4 T cells have equivalent expression of TH1 and TH2 cytokines and lineage markers in SM^+^ and SM^−^ LTBI individuals. PBMCs from the CFP-10 and ESAT-6 stimulated condition were restimulated on day 5 with PMA and ionomycin for 5 h to induce cytokine production. Samples meeting the criteria for a positive proliferative response (see Materials and Methods) were evaluated for cytokine production and expression of lineage specific transcription factors and chemokine receptors by flow cytometry (SM^−^, *n* = 10; SM^+^, *n* = 11). **(A)** Representative flow plots from an SM^+^ LTBI individual. Unstimulated samples (upper) show cytokine production and phenotypes on cells gated on live CD3^+^CD4^+^CD8^−^ lymphocytes. CFP-10/ESAT-6-stimulated samples (lower) show cytokine production and phenotypes on cells gated on live OG^lo^CD3^+^CD4^+^CD8^−^ lymphocytes. **(B)** Frequency of TH1 cytokine^+^ and TH2 cytokine^+^ cells amongst proliferating CD4 T cells. **(C)** Frequency of transcription factor^+^ and chemokine receptor^+^ cells amongst proliferating CD4 T cells. Boxes represent the median and interquartile ranges; whiskers represent the 1.5*IQR. Differences in the frequency of each CD4 T cell population between SM^+^ and SM^−^ individuals were assessed using a Mann Whitney *U*-test.

We next assessed the lineage state of CD4 T cells that proliferate in response to SEB and Mtb peptides. On day 5 of the proliferation assay, PMA and ionomycin was added to OG-labeled PBMCs for 5 h to induce cytokine expression, and the cells evaluated by flow cytometry (Figures 7A and Figure S5A). SEB stimulation induced proliferating CD4 T cells with robust cytokine production capacity, dominated by TH1 cytokines IFNγ and TNFα. The frequencies of these cells did not differ between SM^+^ and SM^−^ individuals in any Mtb infection group (Figure S5B). Proliferating CD4 T cells were also evaluated for their expression of T-bet, GATA3, CXCR3, and CCR4 (Figure S5C). After stimulation for 5 days with SEB, proliferating CD4 T cells expressed high levels of T-bet, GATA3, and CCR4. The frequency of T-bet^+^ proliferating CD4 T cells was higher than the frequency of GATA3^+^ CD4 T cells, whereas the frequency of proliferating CCR4^+^ CD4 T cells was higher than the frequency of CXCR3^+^ CD4 T cells (Figure S5C). None of the markers differed in expression between SM^+^ and SM^−^ individuals. Together these data indicate that there are no intrinsic differences in the lineage phenotypes of proliferating of CD4 T cells attributable to SM infection.

We then evaluated the expression of lineage specific cytokines and phenotypic markers in proliferating Mtb-specific CD4 T cells (Figure 7A). Within the LTBI group, there were similar frequencies of cytokine^+^ proliferating Mtb-specific CD4 T cells between SM^−^ and SM^+^ individuals (Figure 7B). Boolean analysis of cytokine^+^ CD4 T cells indicated that proliferating Mtb-specific CD4 T cells were dominated by IFNγ^+^TNFα^+^ cells (Figure S6A). Proliferating Mtb-specific CD4 T cells expressed high levels of T-bet and moderate levels of GATA3 (Figure 7C). There were also moderate frequencies of CXCR3^+^ and high frequencies of CCR4^+^ proliferating cells. Expression of these four markers was also measured utilizing a Boolean strategy. Interestingly, in contrast to the overnight ICS assay where most Mtb-specific cells were CXCR3^+^ and/or CCR4^+^ but negative for transcription factors, a majority of proliferating Mtb-specific CD4 T cells expressed at least one transcription factor. There was also a substantial fraction of cells that expressed both T-bet and GATA3 (Figure S6B). These data indicate that while Mtb-specific CD4 T cells predominantly produce TH1 cytokines, they are diverse with regards to their expression of TH1 and TH2 lineage markers.

### IFNγ Correlates More Strongly With Both TH1 and TH2 Cytokines in SM^+^ Than SM^−^ LTBI Individuals

**Figure 8:**
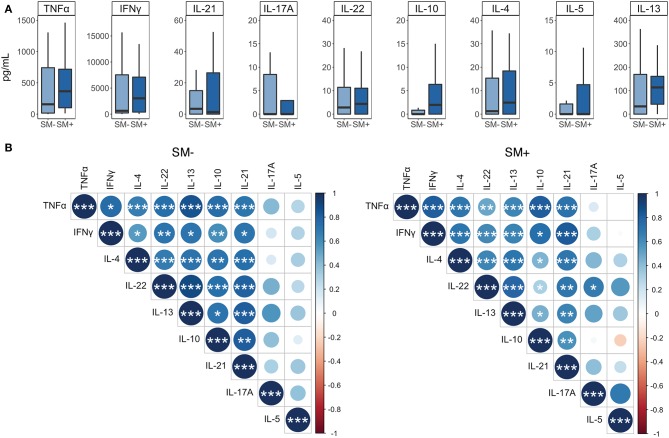
IFNγ is more strongly correlated with the production of additional cytokines in SM^+^ than in SM^−^ LTBI individuals. Supernatants were collected from PBMCs incubated for 5 days under the following conditions: media alone (negative control) or CFP-10 and ESAT-6 peptide pools. **(A)** Levels of each cytokine were quantified by Luminex (SM^−^, *n* = 22; SM^+^, *n* = 22). Data are shown after background subtraction of the respective cytokine in the negative control condition. Boxes represent the median and interquartile ranges; whiskers represent the 1.5*IQR. Differences in the amount of each cytokine between SM^+^ and SM^−^ individuals were assessed using a Mann Whitney *U*-test. **(B)** Correlogram plots of each cytokine, separated by SM infection status. Correlations were evaluated using a non-parametric Spearman rank correlation. Positive correlations are displayed in blue and negative correlations in red. Color intensity and the size of the circle are proportional to the correlation coefficients. Cytokines within each plot were ordered using the centroid method of hierarchical clustering within the SM^−^ data. This order was then applied to the SM^+^ data. ***p < 0.001; **p < 0.01; *p < 0.05.

We next used a Luminex assay to measure cytokine production in PBMC culture supernatants from the 5 days proliferation assay. This allowed us to evaluate a broader range of cytokines, representing multiple T helper subsets, than the ICS assay which was limited to 3–4 cytokines. We collected supernatants on day 5 following stimulation with Mtb CFP-10/ESAT-6 peptide pools, just prior to the addition of PMA and ionomycin. We then measured cytokines associated with TH1 (IFNγ, TNFα), TH2 (IL-4, IL-13, IL-5), TH17 (IL-17A, IL-22), and T-regulatory (IL-10) responses. All nine cytokines were produced at comparable levels between SM^+^ and SM^−^ individuals to Mtb peptides (Figure 8A) as well as to SEB (data not shown).

We also performed correlation analysis to determine which cytokines were most strongly associated with one another. We separated this analysis by SM infection to further evaluate whether SM infection modulates the relationship between different cytokines produced in response to Mtb CFP-10/ESAT-6 peptides. IL-5 did not correlate significantly with any other cytokine, and IL-17A correlated significantly with IL-22, but only in the SM^+^ group. The remaining cytokines all displayed significant positive correlations with each other, many of which differed between SM groups (Figure 8B). In particular, the relationship of IL-10 and IFNγ to the remaining cytokines differentiated these two groups. In SM^−^ individuals, IL-10 correlated more strongly with IFNγ, IL-4, and IL-22, whereas in SM^+^ individuals, IFNγ correlated more strongly with TNFα, IL-4, IL-13, IL-21, and IL-22 (Figure 8B). Together, these data suggest that while individual cytokine levels are similar between SM^+^ and SM^−^ individuals, SM infection modifies the relationship between different cytokines produced in response to Mtb peptide stimulation, particularly with regards to the TH1 cytokine IFNγ.

## Discussion

Infections with a variety of helminths have been shown to dysregulate Mtb-specific CD4 T cell immunity (19–21), however a thorough analysis of CD4 T cell lineage commitment during co-infection with SM and Mtb in humans has not been conducted. We hypothesized that SM infection would modulate the TH1 vs. TH2 lineage profile of Mtb-specific CD4 T cell responses. While there are numerous CD4 T cell subsets, we focused on TH1 and TH2 cells because of their known associations with Mtb and helminth infections, respectively. We found that TH1 cytokine responses are preserved in both the total and Mtb-specific CD4 T cell compartment of SM^+^ and SM^−^ individuals. Moreover, SM^+^ individuals with LTBI had significantly higher frequencies of Th1 cytokine^+^ Mtb-specific CD4 T cells, compared with SM^−^ individuals with LTBI. We also provide evidence that TH1 cytokine^+^ CD4 T cells are flexible in their expression of lineage markers irrespective of Mtb and SM infection status. Lastly we found that SM modulates the lineage expression profile of TH1 cytokine^+^ CD4 T cells in individuals with TB, but not LTBI.

Our data indicate that there are limited differences in the TH1 and TH2 profiles of total CD4 T cells attributable to SM infection. We observed fewer circulating TH1 cells, defined as T-bet^+^ CD4 T cells, in SM^+^ healthy controls than in SM^−^ healthy controls. However, this difference was not observed in the LTBI and TB groups, perhaps due to increased immune activation in Mtb infected individuals at baseline (2, 53, 54). We also observed higher frequencies of circulating CCR4^+^ CD4 T cells in SM^+^ TB individuals, consistent with a previous study indicating elevated CCR4 expression on CD4 T cells in individuals with active TB (55). Although CCR4 is predominately expressed on TH2 cells, it can be expressed on CD4 T cell subsets other than TH2 (56). Lastly, upon mitogen stimulation, the CD4 T cell cytokine profiles of SM^+^ and SM^−^ individuals did not differ in any of the three Mtb infection groups, thus providing further evidence that SM does not globally dysregulate TH1 and TH2 CD4 T cell subsets. Interestingly, there appeared to be a trend toward differences in lineage specific phenotype by Mtb infection status, with CD4 T cells from individuals with active TB having generally lower Th1 cytokine production capacity (Figure 2) and a trend toward higher expression of CCR4 and lower expression of CXCR3 on Mtb-specific CD4 T cells, compared with indivdiuals with LTBI (Figures 4, 5). This suggests that Mtb infection may have a dominant influence over SM infection regarding TH1/TH2 phenotypes of CD4 T cells in peripheral blood. Further studies would need to be conducted to directly evaluate TH lineage phenotype differences by Mtb infection and disease status.

Our findings also provide strong evidence that CD4 T cells are flexible in their expression of lineage specific phenotypic markers. We examined TH1 and TH2 cells, as defined by cytokine production, for the expression of canonical TH1 and TH2 transcription factors and chemokine receptors. T-bet and GATA3 were almost exclusively expressed in TH1 cytokine^+^ and TH2 cytokine^+^ CD4 T cells, respectively and therefore did indeed stratify by functional CD4 T cell subset. By contrast, CXCR3 and CCR4 were expressed on both TH1 cytokine^+^ and TH2 cytokine^+^ CD4 T cells. In addition, while TH2 cytokine^+^ CD4 T cells were predominantly GATA3^+^ and/or CCR4^+^, TH1 cytokine^+^ CD4 T cells were often T-bet^−^ and/or CXCR3^−^. Taken together, this suggests that defining CD4 T cell subsets based on either function or phenotype is not sufficient and does not capture the full variability of these cells. Importantly, this variability is not explained by different immune states of the host since this phenomenon is not different between SM^+^ and SM^−^ individuals, nor between Mtb infection groups. There is growing appreciation that CD4 T cells do not strictly segregate into classical CD4 T cell subsets. Indeed, numerous studies in humans have observed co-production of cytokines as well as co-expression of transcription factors from multiple CD4 T cell lineages at the single cell level (23, 57–61). In many cases, these intermediate cells are associated with differential disease states. Our data supports the concept of CD4 T cell plasticity in humans, however the functional impact of this plasticity requires further study.

Importantly, lineage diversity was observed in Mtb-specific CD4 T cell responses in both LTBI and TB groups. Both groups had TH1 cytokine^+^ Mtb-specific CD4 T cells that expressed not only TH1 but also TH2 lineage markers. Although there was a bias toward T-bet^+^ cells in the overnight assay, GATA3^+^ cells were also detected in the Mtb-specific CD4 T cell repertoire. Furthermore, in both SM^+^ and SM^−^ individuals in the LTBI group, proliferating Mtb-specific CD4 T cells expressed both TH1 and TH2 markers and expressed TH1 cytokines upon restimulation with PMA and ionomycin. This is consistent with previous studies which have reported of co-expression of lineage-specific transcription factors and transcriptional profiles in Mtb-specific CD4 T cells (23, 60, 62). Due to the low frequency of Mtb-specific proliferating CD4 T cells in TB group, consistent with previous reports (52), we were not able to evaluate TH1 and TH2 profiles of proliferating Mtb-specific CD4 T cells in individuals with active TB.

Our data also indicate that the impact of SM infection on Mtb-specific CD4 T cells is dependent on the Mtb infection status of the individual. Contrary to our initial hypothesis, we found that SM^+^ LTBI individuals had higher frequencies of Mtb-specific TH1 cytokine producing CD4 T cells than SM^−^ LTBI individuals. This difference was due to a subset of cells co-producing both TNFα and IFNγ, indicating that SM infection is associated with higher levels of polyfunctional TH1 cells. This differs from studies of co-infection with filarial worms and soil-transmitted helminths, which have reported lower Mtb-specific TH1 cytokine production and lower frequencies of Mtb-specific TH1 cytokine^+^ CD4 T cells in LTBI individuals (18–21). These differences may be due to differences in the life cycles of the worms, especially considering that the lung migrating stage of SM is TH1 stimulating (31). Amongst TB individuals, however, SM^+^ and SM^−^ individuals did not differ in the frequency of Mtb-specific TH1 cytokine^+^ cells. Again this is in contrast to most published studies which report lower TH1 responses in active TB individuals co-infected with either filarial worms or soil-transmitted helminths (15, 21). Interestingly, SM^+^ active TB individuals did have more GATA3^+^CCR4^+^ TH1 cytokine^+^ Mtb-specific cells than SM^−^ active TB individuals. This is consistent with a recent study in Tanzania which reported mixed TH1/TH2 phenotypes in Mtb-specific CD4 T cells in individuals with active TB and helminth coinfection, although the participants in this study were not stratified by helminth species (23). It is difficult to determine whether this is due to TH1 cytokine^+^ cells being skewed to a TH2 phenotype or whether phenotypically TH2 cells are being reprogrammed to produce TH1 cytokines. It has been shown that environmental cues can override programing inherent to lineage specific transcription factors thus providing immunity as needed (63).

The interpretation of our results is limited by cross-sectional enrollment with quota-based sampling. The order of SM and Mtb infection in these individuals may play a role in how CD4 T cells are polarized and respond to subsequent stimuli, although we are not able to confirm that order of SM and Mtb infection in our study cohorts. Infection with SM has been observed in children as young as 3 years old and reaches a prevalence rate of 60% in children 11–13 years old (64, 65). Mass Drug Administration to treat SM infection in school-aged children in Kenya has been successful in reducing prevalence and intensity of SM infection, however reinfection still readily occurs (66). Furthermore, individuals defined as negative for SM infection may have had a previous SM infection are therefore not necessarily SM naïve. This may account for the lack of TH2 cytokine bias observed in the total CD4 T cell population. In addition, while we were able to exclude participants infected with soil transmitted helminths, we did not test for current filarial infections in our study cohort. Although lymphatic filariasis is not endemic in western Kenya (11), other filarial infections have been shown to modulate host immunity (67, 68) and could also influence TH1 vs. TH2 cell differentiation profiles of Mtb-specific CD4 T cell responses. Another limitation of our enrollment design is the inability to compare CD4 T cell profiles in SM^+^ individuals before and after treatment for schistosomiasis. Studies in which individuals were treated for helminth infections have reported increased levels of Mtb-specific chemokines and cytokines following treatment (14, 16, 18, 22). Our analysis was also confined to CD4 T cells specific to the Mtb antigens CFP-10 and ESAT-6, which are immunodominant Mtb antigens known to elicit robust IFNγ responses (69). CD4 T cell responses to other antigens in Mtb may have different cytokine profiles and may be more or less malleable to a TH2 stimulus such as a helminth co-infection. Futhermore, our analysis was limited to evaluation of CD4 T cells circulating in peripheral blood. It is possible that there is more pronounced TH2 skewing of CD4 T cells at the site of Mtb infection in the lung, which has been observed in mice co-infected with Mtb and SM (32). Lastly, by using flow cytometry to evaluate CD4 T cell lineage profiles, we were limited to specific markers for TH1 and TH2 lineage commitment. Future studies, using RNA-sequencing of Mtb-specific CD4 T cells can provide more comprehensive analysis of the Mtb-specific CD4 T cell repertoire including additional CD4 T cell subsets and functions.

The environment in which immune cells function is vast, diverse and constantly changing. It is of critical importance that CD4 T cells, which orchestrate the immune response to pathogens, commensals, and self, be able to interpret mixed signals and initiate the responses required for survival of the host. Furthermore, they must be able to flexibly respond to changes in the environment, whether they be competing stimuli, changes in antigen load, or changes in tissue structure. We provide evidence that while infection with SM can skew the phenotype of CD4 T cells under certain conditions, it does not compromise the ability of CD4 T cells to mount a functional TH1 response to Mtb. Furthermore, the impact of SM on functional TH1 responses to Mtb depends on the clinical status of the Mtb infection, with co-infected LTBI individuals actually having higher Mtb-specific TH1 cytokine responses than those with LTBI alone. When considering that pathogens such as Mtb and SM are co-endemic in many areas of the world, this flexibility in the immune system is highly advantageous to the host in being able to mount immune responses to multiple different pathogens simultaneously.

## Data Availability Statement

The datasets generated for this study are available on request to the corresponding author.

## Ethics Statement

The studies involving human participants were reviewed and approved by KEMRI/CDC Scientific and Ethics Review Unit and Emory University Institutional Review Board. The patients/participants provided their written informed consent to participate in this study.

## Author Contributions

CD and TM contributed conception and design of the study, contributed to data interpretation, statistical analyses, and drafted the manuscript. TM, JK, JO, and JT performed experimental work. CD, TM, SG, FO, SA, AC, and NG contributed to execution and oversight of experimental work, participant recruitment and enrollment, and study database management. All authors approved the final manuscript.

### Conflict of Interest

The authors declare that the research was conducted in the absence of any commercial or financial relationships that could be construed as a potential conflict of interest.
