# Supplementary material for: CD4 T Cells in Mycobacterium tuberculosis and Schistosoma mansoni Co-infected Individuals Maintain Functional TH1 Responses
Source: Front Immunol. 2020 Feb 7;11:127. doi: 10.3389/fimmu.2020.00127 (PMC7020828; doi:10.3389/fimmu.2020.00127)
Supplement: Supplementary file 1 [file Data_Sheet_1.PDF]

Supplementary Material

CD4 T cells in *Mycobacterium tuberculosis* and *Schistosoma mansoni* co-infected individuals maintain functional TH1 responses

Taryn A. McLaughlin, Jeremiah Khayumbi, Joshua Ongalo, Joan Tonui, Loren E. Sasser, Angela Campbell, Salim Allana, Samuel Gurrion Ouma, Felix Hayara Odhiambo, Neel R. Gandhi, Cheryl L. Day*

***Correspondence:** Cheryl L. Day: cday@emory.edu

1. Supplementary Figures

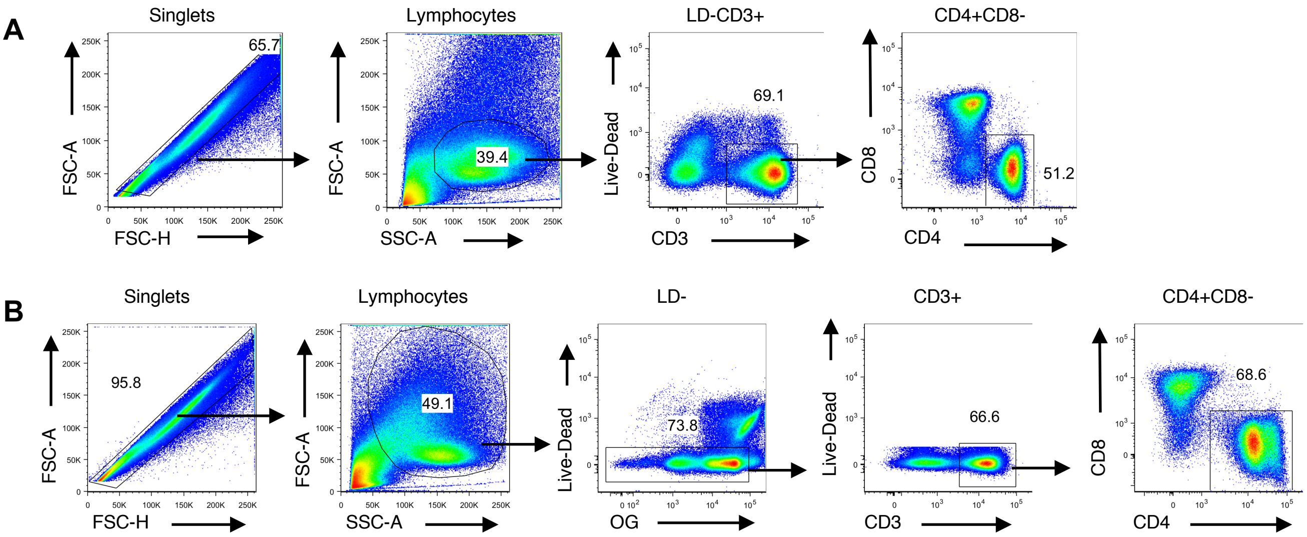

Figure S1. Gating strategy for flow cytometry analysis. **(A)** In this sample gating for the overnight ICS assay, cells were first gated for singlets (FSC-H vs. FSC- A) and lymphocytes (SSC-A vs. FSC-A). The lymphocyte gate is further analyzed for their uptake of the Zombie IR Live/Dead stain to determine live versus dead cells and their expression of CD3 (Zombie Near-IR^{lo}, CD3^{+}). CD4 and CD8 surface expression is then determined from this gated population. **(B)** In this sample gating for the Proliferation ICS assay, cells were first gated for singlets (FSC-H vs. FSC- A) and lymphocytes (SSC-A vs. FSC-A). The lymphocyte gate is further analyzed for their uptake of the Zombie IR Live/Dead stain to determine live versus dead cells (Zombie Near-IR^{lo}). Live cells are then gated for their expression of CD3 (CD3^{+}) and CD4 and CD8 surface expression is then determined from this gated population.

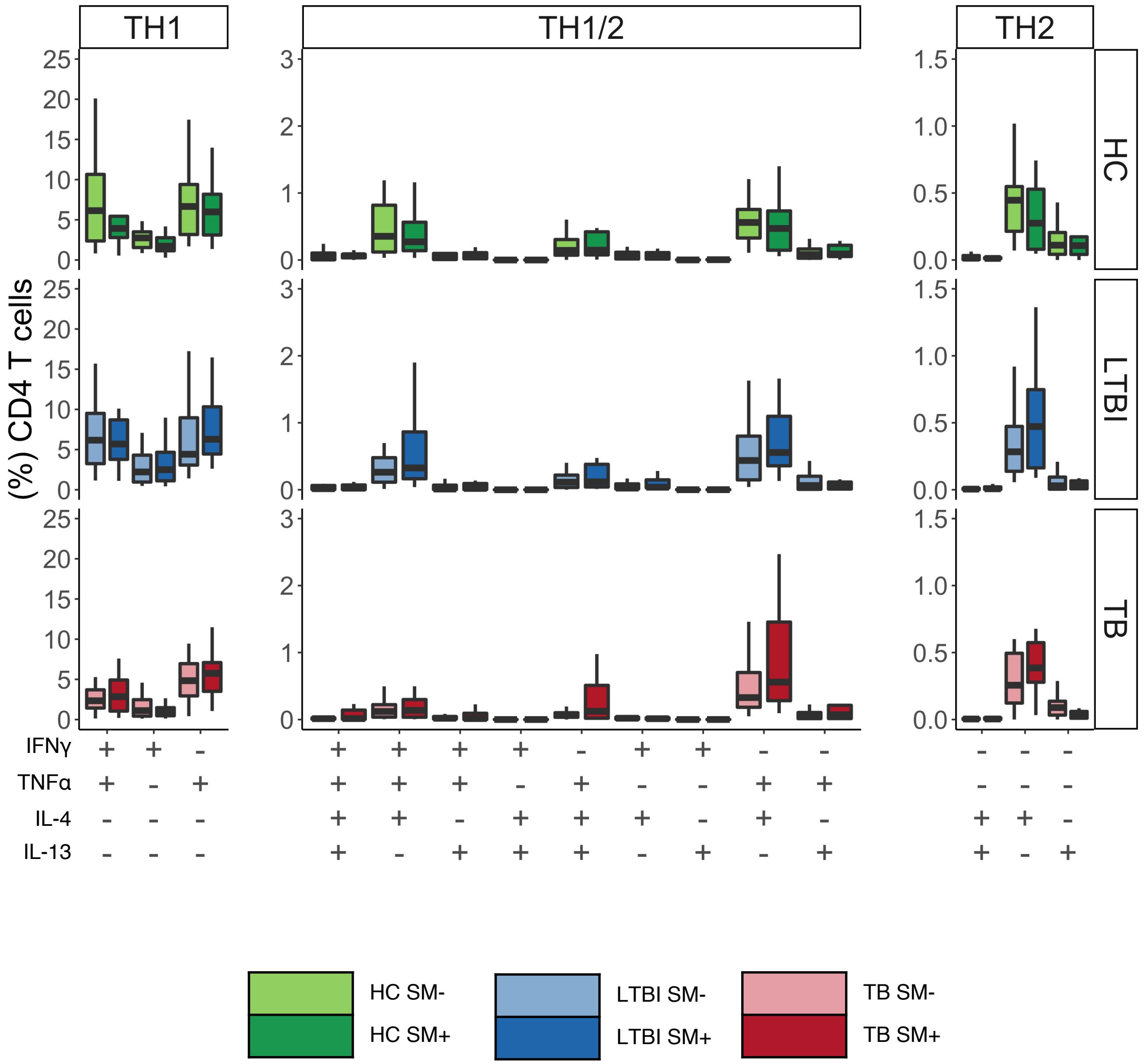

Figure S2. SM⁺ and SM⁻ individuals have similar frequencies of cytokine⁺ CD4 T cells across all combination of TH1 and TH2 cytokines. PBMC samples obtained from individuals in each of six groups defined by TB and *S. mansoni* infection status were incubated for 18 h in media alone (negative control) or stimulated with PMA and Ionomycin. Intracellular expression of IFNγ, TNFα, IL-4 and IL-13 was measured by flow cytometry (HC SM^-, n=24; HC SM^+, n=13; LTBI SM^-, n=25; LTBI SM^+, n=25; TB SM^-, n=25; TB SM^+, n=15). Frequency of each combination of cytokines using a Boolean gating strategy grouped by TH lineage. Data are shown after subtraction of background cytokine production in the unstimulated negative control condition. Boxes represent the median and interquartile ranges; whiskers represent 1.5*IQR. Differences in the frequency of each $cytokine^+$ CD4 T cell population between SM^+ and SM^- individuals were assessed using a Mann Whitney *U* test. P-values < 0.05 were considered significant.

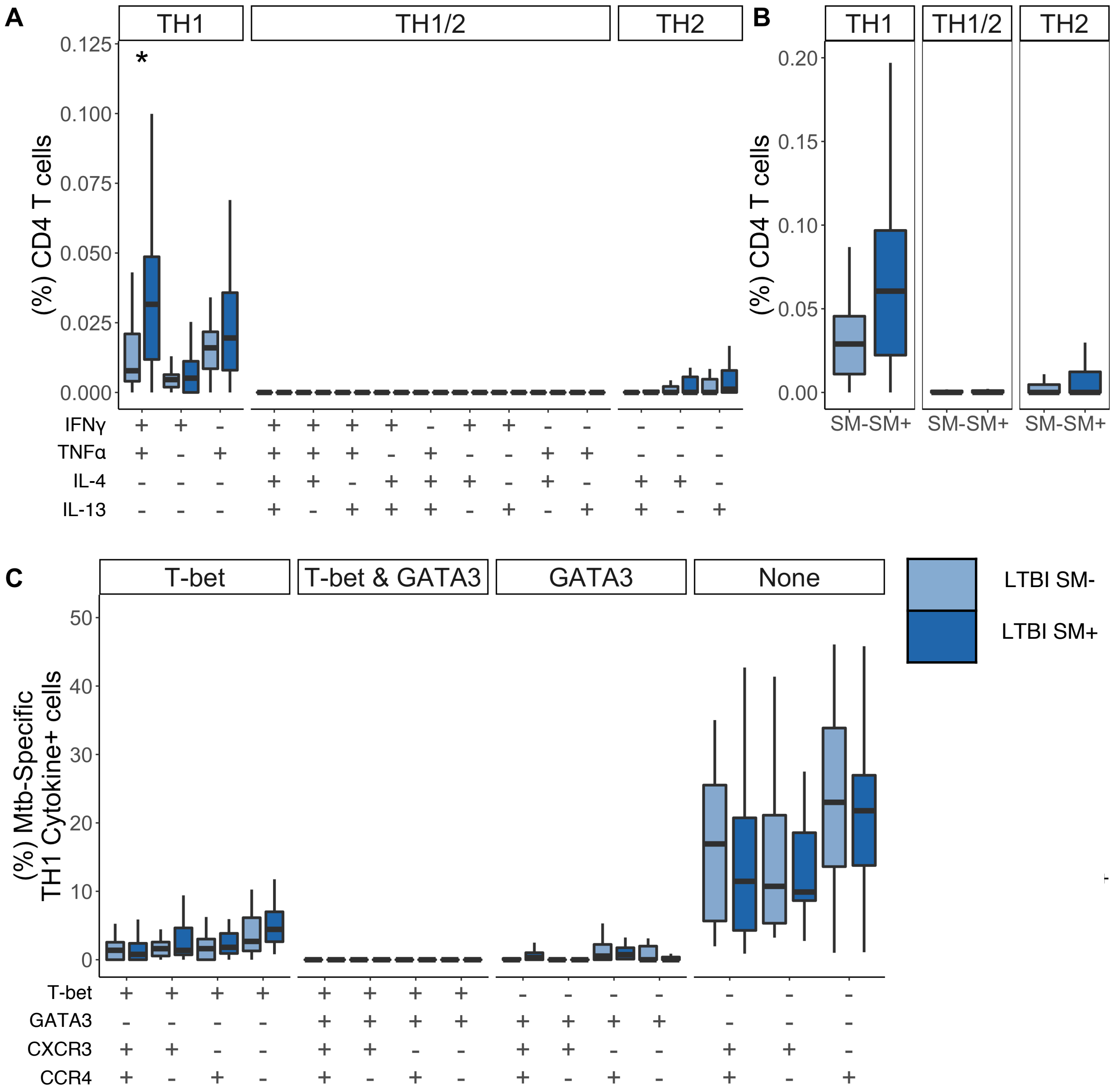

Figure S3. SM$^+$ LTBI individuals have higher frequencies of IFNγ$^+$TNFα$^+$ Mtb-specific CD4 T cells, which express both TH1 and TH2 lineage markers. PBMC samples obtained from SM$^+$ and SM$^-$ LTBI individuals were stimulated for 18 h with Mtb peptides CFP-10 and ESAT-6. Intracellular expression of IFNγ, TNFα, IL-4 and IL-13 was measured by flow cytometry (SM$^-$, n=24; SM$^+$, n=22). (**A**) Frequency of each combination of cytokines using a Boolean gating strategy. (**B**) Frequency of each aggregated group of TH cytokine$^+$ CD4 T cells as defined in S4A. Samples meeting the criteria for a positive response (see Materials and Methods) were evaluated for expression of lineage specific phenotypic markers using a Boolean gating strategy. (**C**) Frequency of each combination of transcription factors and chemokine receptors amongst TH1 cytokine$^+$ CD4 T cells (SM$^-$, n=16; SM$^+$, n=18). Boxes represent the median and interquartile ranges; whiskers represent the 1.5*IQR. Differences in the frequencies of TH1, TH1/2, and TH2 CD4 T cells within each group were evaluated using a Kruskal Wallis test. TH1 cytokine frequencies were statistically higher than the both TH1/2 and TH2 frequencies after applying the Bonferroni correction for multiple comparisons. Differences in the frequency of each CD4 T cell population between SM$^+$ and SM$^-$ individuals were assessed using a Mann Whitney *U* test. **: p< 0.01; *:p< 0.05

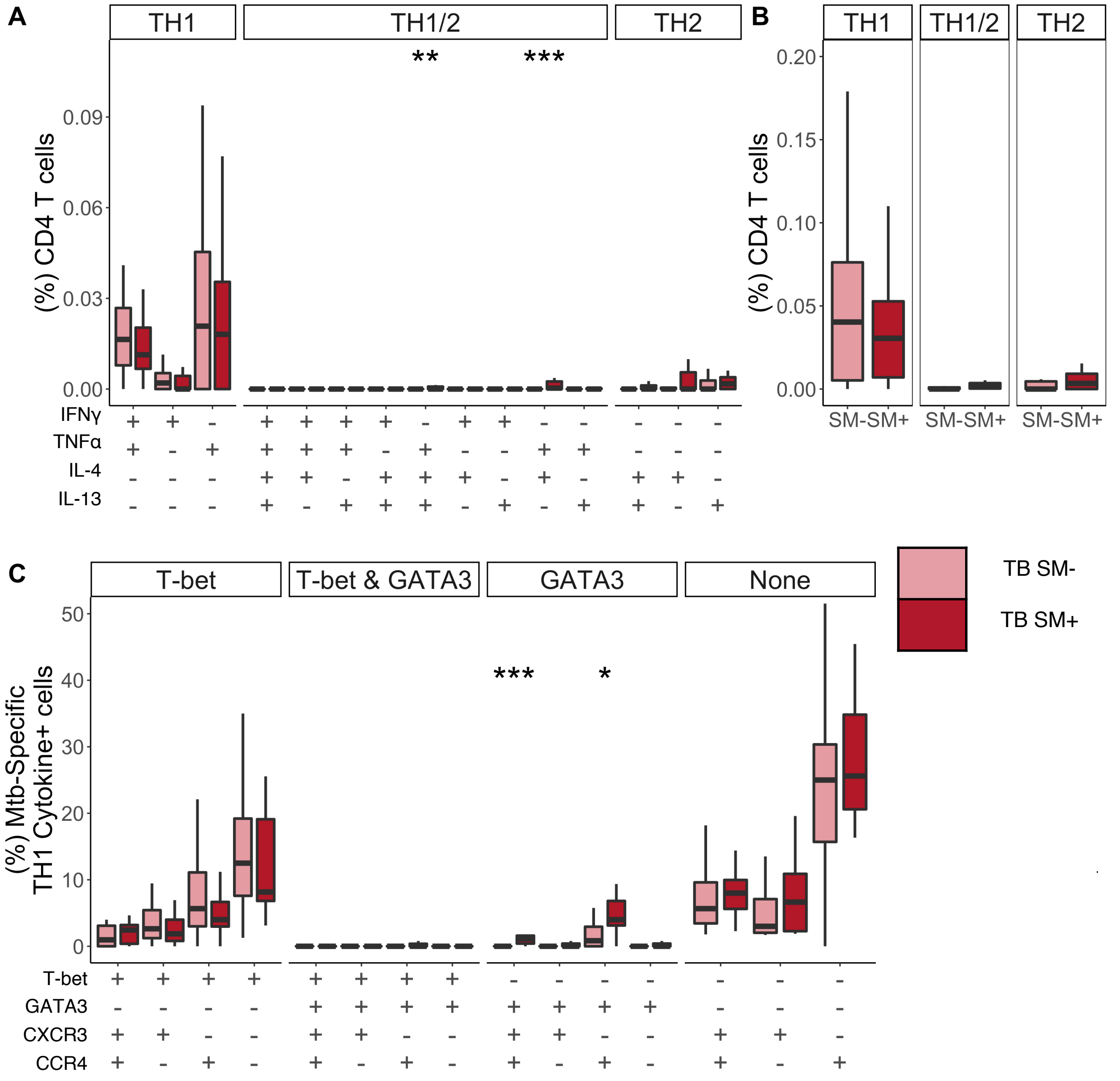

Figure S4. SM⁺ TB individuals have higher frequencies of IL-4⁺TNFα⁺ and GATA3⁺CCR4⁺ Mtb-specific CD4 T cells. PBMC samples obtained from SM⁺ and SM⁻ TB individuals were stimulated for 18 h with Mtb peptides CFP-10 and ESAT-6. Intracellular expression of IFNγ, TNFα, IL-4 and IL-13 was measured by flow cytometry (SM⁻, n=25; SM⁺, n=15). (**A**) Frequency of each combination of cytokines using a Boolean gating strategy. (**B**) Frequency of each aggregated group of TH cytokine⁺ CD4 T cells as defined in S5A. Samples meeting the criteria for a positive response (see Materials and Methods) were evaluated for expression of lineage specific phenotypic markers using a Boolean gating strategy. (**C**) Frequency of each combination of transcription factors and chemokine receptors amongst TH1 cytokine⁺ CD4 T cells (SM⁻, n=15; SM⁺, n=9). Boxes represent the median and interquartile ranges; whiskers represent the 1.5*IQR. Differences in the frequencies of TH1, TH1/2, and TH2 CD4 T cells within each group were evaluated using a Kruskal Wallis test. TH1 cytokine frequencies were statistically higher than the both TH1/2 and TH2 frequencies after applying the Bonferroni correction for multiple comparisons. Differences in the frequency of each CD4 T cell population between SM⁺ and SM⁻ individuals were assessed using a Mann Whitney *U* test. **: $p < 0.01$; *:$p < 0.05$

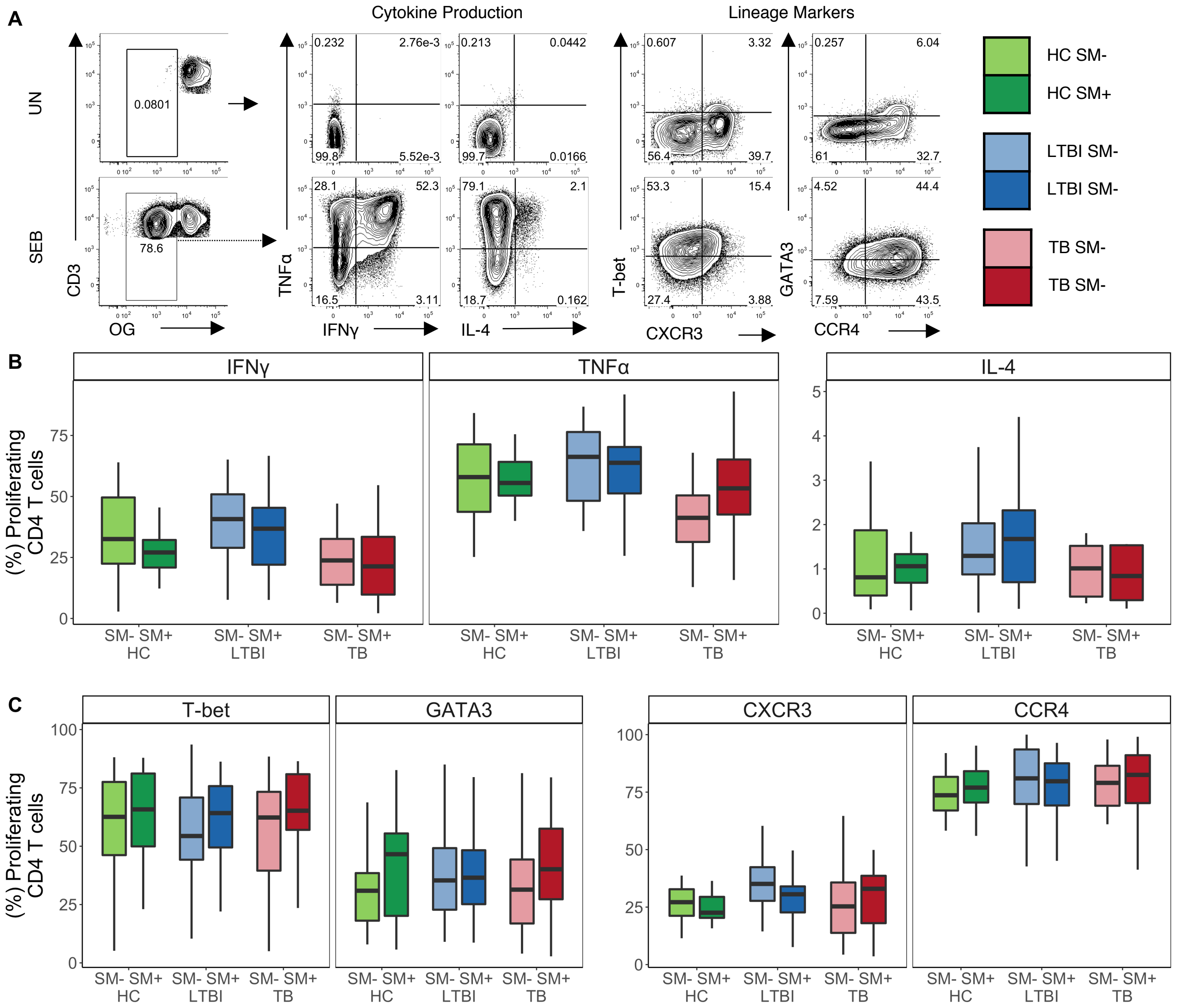

Figure S5. Proliferating CD4 T cells have equivalent expression of TH1 and TH2 cytokines and lineage markers in *S. mansoni*⁺ and *S. mansoni*⁻ individuals across Mtb infection groups. PBMC from SEB stimulated condition were restimulated on day 5 with PMA and Ionomycin for 5 hours to induce cytokine production. Samples meeting the criteria for a positive proliferative response (see Materials and Methods) were evaluated for cytokine production and expression of lineage specific transcription factors and chemokine receptors by flow cytometry. **(A)** Representative flow plots from an *S. mansoni*$^+$ LTBI individual. Unstimulated samples (upper) show cytokine production and phenotypes on cells gated on live $CD3^+CD4^+CD8^-$ lymphocytes. SEB samples (lower) show cytokine production and phenotypes on cells gated on live $OG^{lo}CD3^+CD4^+CD8^-$ lymphocytes. **(B)** Frequency of TH1 cytokine$^+$ and TH2 cytokine$^+$ cells amongst proliferating CD4 T cells. **(C)** Frequency of transcription factor$^+$ and chemokine$^+$ cells amongst proliferating CD4 T cells. Boxes represent the median and interquartile ranges; whiskers represent the minimum and maximum 1.5*IQR. Differences in the frequency of each CD4 T cell population between *S. mansoni*$^+$ and *S. mansoni*$^-$ individuals were assessed using a Mann Whitney *U* test. *P-values* < 0.05 were considered significant.

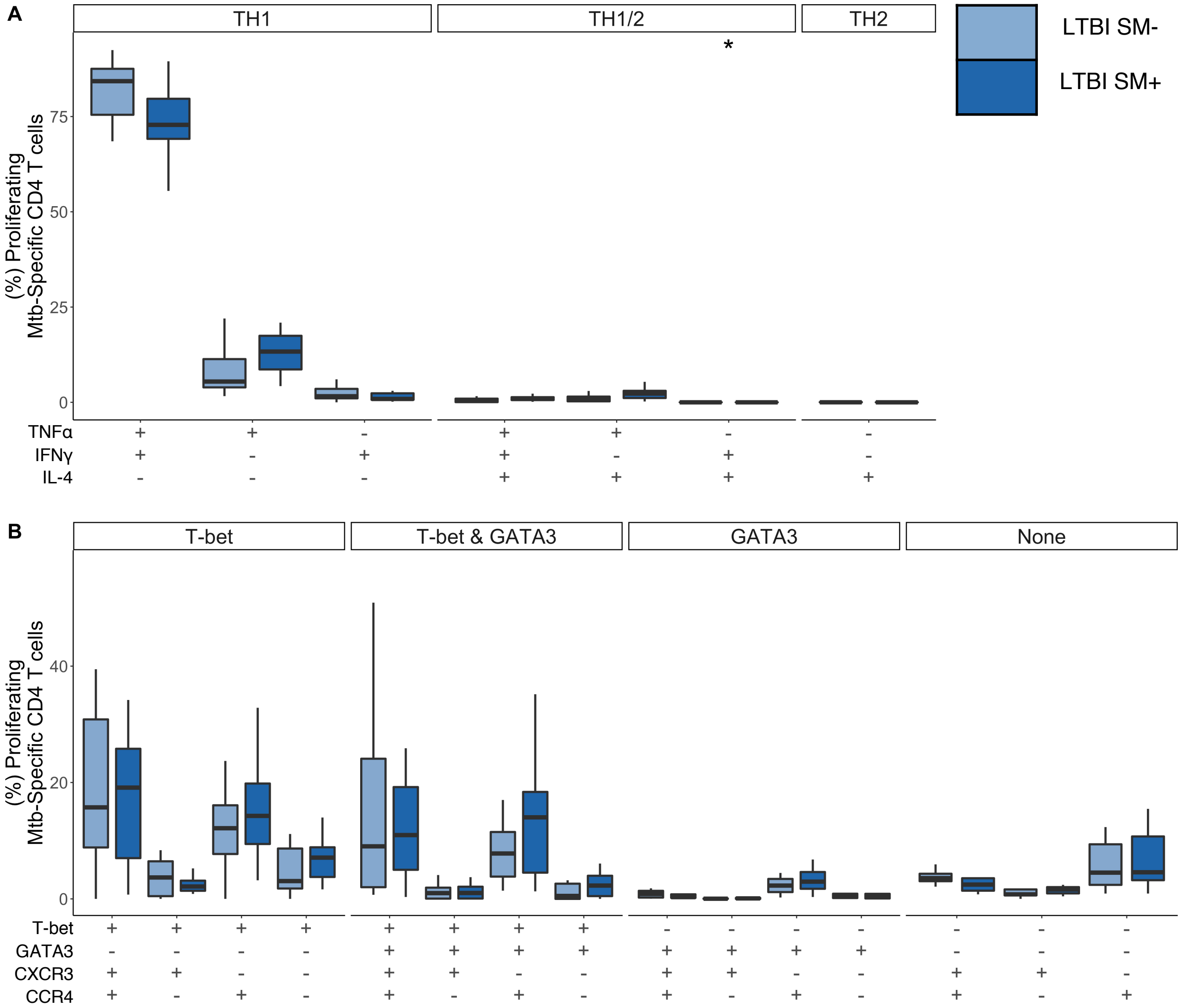

Figure S6. Proliferating Mtb-specific CD4 T cells produce TH1 cytokines and express both TH1 and TH2 lineage markers in SM$^+$ and SM$^-$ LTBI individuals. PBMC from the CFP-10 and ESAT-6 stimulated condition were restimulated on day 5 with PMA and Ionomycin for 5 hours to induce cytokine production. Samples meeting the criteria for a positive proliferative response (see Materials and Methods) were evaluated for cytokine production and expression of lineage specific transcription factors and chemokine receptors by flow cytometry (SM$^-$, n=10; SM$^+$, n=11). **(A)** Frequency of each combination of TH1 and TH2 cytokine$^+$ cells amongst proliferating CD4 T cells. **(B)** Frequency of each combination of transcription factor$^+$ and chemokine receptor$^+$ cells amongst proliferating CD4 T cells. Boxes represent the median and interquartile ranges; whiskers represent the 1.5*IQR. Differences in the frequency of each CD4 T cell population between SM$^+$ and SM$^-$ individuals were assessed using a Mann Whitney *U* test. *:$p < 0.05$
